# Cases of Croup, Communicated by Dr. Maton

**Published:** 1801-06

**Authors:** W. G. Maton

**Affiliations:** Craven Street


### *To the Editors of the Medical and Phyfical Journal.*

GENTLEMEN,

THE following cafes of Cynanche Trachealis were tranfmitted to me by a very ingenious and refpectable practitioner in Buckinghamfhire, for the purpofe of being communicated to a medical fociety in this metropolis. They are certainly worthy of greater publicity than they could have by being fimply read before a confined number of profeffional men, and I therefore requeft the favour of you to infert them in your widely circulated Journal. As I was not defired to publifh this communication, I do not feel myfelf at liberty to mention the name of the author, whofe modefty may render him averfe from appearing in print, and whofe merit will daily accelerate his arrival at eminence without it.

*Craven Street,*
*April* 25, 1801.

I am, &c.
W. G. MATON.

February

February 5, 1801. At ten in the evening I faw the daughter of Colonel S. in this neighbourhood, who awoke from her fleep with a fuffocating ftricture upon her breath, accompanied with the peculiar found characterifing croup. She had during the day been obferved to be hoarfe as from a common cold, but was not otherwife unwell. The mother, having once before witneffed the croup, gave an emetic of antimon. tartaris, which operated powerfully, on the firft alarm; by this the trumpet-like found, and the difficulty of breathing were in a fmall degree diminifhed. But at the time of my firft feeing her, about two hours after fhe had awoke, fhe ftill breathed very laborioufly. The peculiar found, the *suffocatio stridula* was chiefly perceptible on coughing and crying, at which times it was in the higheft degree diftreffing.

I remained with her through the night. In about an hour after my arrival, her diftrefs being extreme in breathing, and the found increafing, I gave her another emetic, by which fhe was in fome meafure relieved. Four leeches were immediately applied to the throat, and their wounds encouraged to bleed by warm fomentations more than an hour and a half. Within a fhort time after they had ceafed to bleed, two blifters of confiderable fize were laid on the throat.

The blifters acted quickly; and by the morning there was a manifeft abatement of the fymptoms, but the breathing was ftill difficult, and by no means free from the found of croup. She had no mark of general illnefs, her fkin being of the natural heat, and pulfe, as far as could be judged, not unufually quick for a child of her age, which was two years and a half, varying confiderably, however, after diftreffing fits of coughing or crying. The bowels were in a natural ftate.

A grain and a half of calomel was ordered to be given once in two hours in fugar, and a drachm of the mild mercurial ointment to be rubbed into the thighs carefully once in four hours. In the afternoon the fymptoms increafed again, and continued to do fo until the morning of the 7th, from which time there was a gradual diminution of them to the evening of the 9th, after which the found of croup was not heard more than two or three times, and then in the act of fcreaming. The breathing became natural, and the cough difappeared. In the whole fhe took forty-eight grains of calomel, and had rubbed in feven drachms of the ointment.

On the morning of the 7th, two other children in the fame family, a boy of five, and a girl of four years old, were feized with fymptoms of croup, which by the evening had increafed to a high degree. Emetics were given, and four leeches, and two blifters applied in each cafe; by thefe, as in the former cafe, cafe, an abatement of the difeafe was obtained, but by no means was it entirely removed. In a few hours after the action of thefe remedies was over, the difficulty of breathing with the ftridulous found became more urgent; and the mercurial plan was begun immediately with both. At the end of three days, the boy had taken 59 grains, and the girl 52 grains of calomel. For the former, 10 drachms; and for the latter, 9 drachms of the *strong* mercurial ointment were rubbed in within the fame fpace of time. Under the ufe of the mercury the difeafe fubfided, and was not obferved in any fhape after the fifth day from the commencement of its employment.

On the 9th, an infant of four months, which had very lately recovered from a diarrhœa, that from its violence and long continuance had threatened to carry it off, was heard feveral times *to croup*; and its breathing became difficult, fo that it could not well fuck. An emetic was immediately given of ipecacuanha, and two leeches were foon after applied to the throat. The leeches ufed for this child were large, and made wounds which did not, in its weak ftate, readily clofe. Notwithftanding many endeavours were made to ftop the bleeding, it continued with little interruption for thirty hours; fometimes it was a mere oozing, at other times it was very free. The child at length became fo exhaufted, that it became neceffary to give it frequently foft gruel with a fmall proportion of wine. The bleeding ceafed during an hour or two of great faintnefs; after which no mark of croup was ever obferved. No other remedy was employed in this cafe. The child in a few days recovered from the effects of the bleeding, and ultimately became quite well.

In the three former of the above cafes, the fymptoms of croup exifted in a highly diftreffing degree; in neither of them was there any conftitutional affection. In all, the effects of the remedies employed were the fame. The force of the difeafe was broken by the firft remedies, and more efpecially, as it appeared, by the emetics, although in none was there any diflodgement of the membranous matter lining the trachea in this difeafe, which has fometimes happened. In all, the difeafe gave way to mercury. In thefe children the mercury produced no purgative nor conftitutional effect whatever; I fhould indeed have doubted if it had been fairly ufed in the quantity related, had I not been prefent during the greater part of their illnefs. The calomel was always entirely fwallowed, and the ointment very diligently rubbed in. The curative effect of mercury in croup has been before eftablifhed in this part of the country, in which the difeafe is not very uncommon; and I believe the difficulty of fo impregnating the fyftem with it as to to produce the common evidences, has been noticed by Dr. Ruſh and others, who have ſeen the utility of that remedy in this moſt diſtreſſing, and moſt rapid in its progreſs, of the diſeaſes to which children are liable. I am not certain, ſince the introduction of mercury into the treatment of croup, whether too little uſe may not have been made of the older remedies. The event of theſe caſes has convinced me that they have a powerful effect in breaking the force of the diſeaſe at its onſet, although they have not in all inſtances a power of entirely deſtroying it. In the three firſt caſes, the ſubjects of which were remarkably healthy, the peculiar inflammation of croup was not ſubdued by them. (It may be a queſtion whether a larger number of leeches, &c. might not have cured the diſeaſe.) In the caſe of the infant, weakened by previous diſeaſe, and weakened now by exceſſive bleeding, no further remedy was required. The old plan, however, has often failed; the mercurial plan never I believe in this neighbourhood.

